# Impact of sex on the adaptation of adult mice to long consumption of sweet-fat diet

**DOI:** 10.18699/VJ20.682

**Published:** 2020-12

**Authors:** N.M. Bazhan, T.V. Iakovleva, A.D. Dubinina, E.N. Makarova

**Affiliations:** Institute of Cytology and Genetics of Siberian Branch of the Russian Academy of Sciences, Novosibirsk, Russia Novosibirsk State University, Novosibirsk, Russia; Institute of Cytology and Genetics of Siberian Branch of the Russian Academy of Sciences, Novosibirsk, Russia; Institute of Cytology and Genetics of Siberian Branch of the Russian Academy of Sciences, Novosibirsk, Russia; Institute of Cytology and Genetics of Siberian Branch of the Russian Academy of Sciences, Novosibirsk, Russia

**Keywords:** C57BL/6J mice, sweet-fat diet, adiposity, sex differences, liver, adipose tissue, FGF21, insulin, gene expression, мыши C57BL/6J, сладко-жирная диета, ожирение, половые различия, печень, жировая ткань, FGF21, инсулин, экспрессия генов.

## Abstract

In rodents, the most adequate model of human diet-induced obesity is obesity caused by the consumption of a sweet-fat diet (SFD), which causes more pronounced adiposity in females than in males. The aim of this work was to determine the sex-associated effect of SFD on the expression of genes related to carbohydrate-lipid metabolism in adult mice. For 10 weeks, male and female С57Bl mice were fed a standard laboratory chow (Control group) or a diet, which consisted of laboratory chow supplemented with sweet cookies, sunflower seeds and lard (SFD group). Weights of body, liver and fat depots, blood concentrations of hormones and metabolites, liver fat, and mRNA levels of genes involved in regulation of energy metabolism in the liver, perigonadal and subcutaneous white adipose tissue (pgWAT, scWAT) and brown adipose tissue (BAT) were measured. SFD increased body weight and insulin resistance in mice of both sexes. Female mice that consumed SFD (SFD females) had a greater increase in adiposity than SFD males. SFD females showed a decreased expression of genes related to lipogenesis (Lpl) and glucose metabolism (G6pc, Pklr) in liver, as well as lipogenesis (Lpl, Slca4) and lipolysis (Lipe) in pgWAT, suggesting reduced energy expenditure. In contrast, SFD males showed increased lean mass gain, plasma insulin and FGF21 levels, expressions of Cpt1α gene in pgWAT and scWAT and Pklr gene in liver, suggesting enhanced lipid and glucose oxidation in these organs. Thus, in mice, there are sex-dependent differences in adaptation to SFD at the transcriptional level, which can help to explain higher adiposity in females under SFD consumtion.

## Abstract (ru)

Наиболее адекватной моделью диет-индуцированного ожирения у человека является ожирение грызунов, вызванное потреблением сладко-жирной диеты (СЖД), которая в большей степени увеличивает долю жира у женщин, чем у мужчин. Целью работы было определение обусловленного полом влияния СЖД на экспрессию генов, контролирующих углеводно-жировой обмен у взрослых мышей. Самцов и самок мышей линии C57BL/6J кормили в течение 10 недель стандартной лабораторной пищей (контрольная группа) или диетой, которая состояла из лабораторной пищи с добавлением сладкого печенья, семян подсолнечника и сала (группа СЖД). Были измерены вес тела, печени и жировых депо, концентрация гормонов и метаболитов в крови, содержание жира в печени и уровни мРНК генов, участвующих в регуляции энергетического обмена, в печени, окологонадном и подкожном белом жире и в буром жире. Потребление СЖД вызвало ожирение (у самок в большей степени, чем у самцов) и резистентность к инсулину у мышей обоих полов. У самок, получавших СЖД, была снижена относительно контроля экспрессия генов печени, связанных с липогенезом (Lpl), метаболизмом глюкозы (G6pc, Pklr), и генов белого жира, связанных с липогенезом (Lpl, Slca4) и липолизом (Lipe), что предполагает снижение расхода энергии в этих тканях. Потребление СЖД у самцов, в отличие от самок, резко повысило уровни в крови инсулина и FGF21, а также экспрессию гена Cpt1α в окологонадном и подкожном белом жире и гена Pklr в печени, что свидетельствует об усилении окисления липидов и глюкозы в этих тканях. Таким образом, у мышей были выявлены половые различия в адаптации к СЖД как на уровне целого организма, так и на уровне транскрипции генов. Эти результаты могут способствовать развитию поло-специфических подходов к коррекции ожирения у человека.

## Introduction

In the human population, there is a significant increase in the number of people suffering from obesity and associated metabolic diseases such as type 2 diabetes, cardiovascular diseases and non-alcoholic fatty liver. The mechanisms of obesity development are studied in laboratory animals with various models of diet-induced obesity. Among the high-calorie diets, the high-fat and the sweet-fat diet (SFD), or the cafeteria diet are the most popular. SFD is most consistent with the consumption of “pleasant” food, which provokes the development of obesity in the human population (Sampey et al., 2011). A special study carried out on male rats showed that SFD more effectively than a high-fat diet induced the development of obesity, hyperphagia, and increased blood cholesterol and leptin levels (Buyukdere et al., 2019).

It is known that most of the characteristics of energy metabolism differ in males and females (Mauvais-Jarvis, 2015). However, the question of the impact of sex on the adaptation of adult mice to long-term consumption of a SFD remains unexplored.

Fibroblast Growth Factor 21 (FGF21) is a protein hormone of the liver that helps the body adapt to metabolic stresses (hunger, cold, overeating and obesity) (Fisher et al., 2010). Exogenous FGF21 reduces body weight, normalizes the lipid profile, and increases insulin sensitivity in various models of obesity and insulin resistance (Zhang, Li, 2014). Earlier, we and others showed that, SFD dramatically increased blood FGF21 level and its hepatic gene expression in mature male, but not female mice (Chukijrungroat et al., 2017; Gasparin et al., 2018; Bazhan et al., 2019). Based on this, it can be assumed that adult males and females will differ in the ways of adaptation to the consumption of SFD. The effects of FGF21 are partially realized through the regulation of the expression of genes controlling carbohydrate-lipid metabolism in the liver, white and brown fat (Coskun et al., 2008; Camporez et al., 2013). The aim of this work was to study the ways of adaptation to the consumption of SFD at the level of the whole organism and at the level of expression of genes involved in lipid and carbohydrate metabolism in the liver and adipose tissue, in mature male and female mice.

## Materials and methods

All experiments were performed according to the European Convention for the Protection of Vertebrate Animals used for Experimental and other Scientific Purposes (Council of Europe No. 123, Strasbourg, 1985) and Russian national instructions for the care and use of laboratory animals. The protocols were approved by the Independent Ethics Committee of the Institute of Cytology and Genetics of the Siberian Branch of the Russian Academy of Sciences.

Animals. Ten-week-old C57BL mice (the vivarium of the Institute of Cytology and Genetics) were used. Both male and female mice were housed in group (3 mice per cage) and were fed with standard laboratory chow (Assortiment Agro, Moscow region, Turacovo, Russia) (control diet, control) or with mixed diet, which consisted of standard laboratory chow supplemented with sweet cookies, sunflower seeds and lard (sweet-fat diet, SFD). There were 4 experimental groups (5–7 mice per group): control male, control female, SFD male and SFD female.

Mice were killed by decapitation after 10 weeks of diet, liver, white adipose tissue (WAT) of different localizations (perigonadal, pgWAT, subcutaneous, scWAT, and perirenal), and interscapular brown adipose tissue (BAT) were weighed. Lean body weight was determined by subtracting the total fat mass from the body weight. Gene expression was measured in the samples of these tissues, excluding perirenal WAT.

Assay of plasma biochemical parameters. Trunk blood was collected in test tubes with EDTA after decapitation, centrifuged and plasma was stored at –20 °C until the assay of hormones and metabolites. Concentrations of FGF21, insulin, adiponectin, and leptin were measured using the following ELISA Kits: Rat/Mouse Fibroblast Growth Factor-21 ELISA Kit, Rat/Mouse Insulin ELISA Kit, Mouse Adiponectin ELISA Kit и Mouse Leptin ELISA Kit (Millipore, St. Louis, MI, USA). Concentrations of glucose, free fatty acids (FFA), triglycerides (TG), and cholesterol were measured colorimetrically using Fluitest GLU, Fluitest TG, Fluitest CHOL (Analyticon Biotechnologies AG, Lichtenfels, Germany) and NEFA FS kits (non-esterified fatty acids) (DiaSys, Germany).

Glucose tolerance and insulin tolerance tests. On the day of testing, the animals were removed from the food at 10:00 am, and the water was left ad libitum. Insulin tolerance test (ITT) started at 2:00 pm, glucose tolerance test (GTT) – at 4:00 pm. In GTT, a glucose solution in water at a dose of 2 mg/1 g of body weight was administered orally. In the ITT, animals were injected intraperitoneally with protofan in physiological saline at a dose (0.5 IU/1 kg of body weight). The glucose level was determined in the blood from the tail vein using test strips and a OneTouch Select glucometer (Lifescan; Johnson and Johnson, USA) before drug administration and 15, 30, 60, and 120 minutes after administration in GTT and after 15, 30, 60 minutes in ITT.

The reaction of reverse transcription and real-time PCR. Total RNA was isolated from tissue samples with ExtractRNA (Evrogen, Moscow, Russia) according to the manufacturer’s instructions. First-strand cDNA was synthesized with Moloney murine leukemia virus (MMLV) reverse transcriptase (Evrogen, Moscow, Russia) and oligo (dT) as a primer. Applied Biosystems TaqMan Gene Expression Assays, listed in Table 1, and qPCRmix-HS LowROX Master Mix (Evrogen, Moscow, Russia) were used for relative quantitation real-time PCR with β-actin as an endogenous control. Sequence amplification and fluorescence detection were performed with the Applied Biosystems ViiA™ 7 Real-Time PCR System (Life Technologies, 5791 Van Allen Way, Carlsbad, CA, USA). Relative quantitation was performed by the comparative CT method, where CT is the cycle threshold.

**Table 1.**
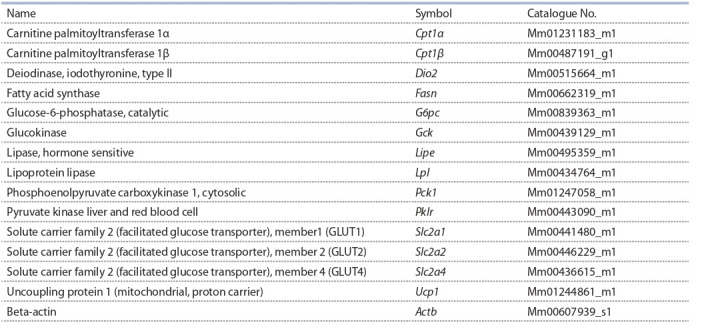
Taqman gene expression assays for mice (Applied Biosystems)

Statistical analysis. The results are presented as means±SE from the indicated number of mice. Two-way ANOVA with factors sex (male, female) and diet (standard diet, control group and sweet and fat diet, SFD group) was used to analyze effect of sex and SFD on blood parameters, gene expression and area under curves in GTT and ITT with multiple comparisons using the post hoc Tukey test. Three-way ANOVA with factors sex, diet, and time (minutes 0, 15, 30, 60, 120 for GTT and 0, 15, 30, 60 for ITT) was used to analyze the results of GTT and ITT. Where indicated, groups were also compared using Student’s t-test. Significance was determined as p < 0.05. The STATISTICA 6 software package (StatSoft, USA) was used for analysis.

## Results

Weight characteristics

In females, body weight was lower than in males in both groups (P < 0.001) (Table 2). Under the SFD, both male and female mice gained more weight than their respective control diet fed counterpart (P < 0.001). FD consumption increased body weight: in males – by 39 %, and in females – by 40 % and contributed to the maximum manifestation of sex differences.

**Table 2.**
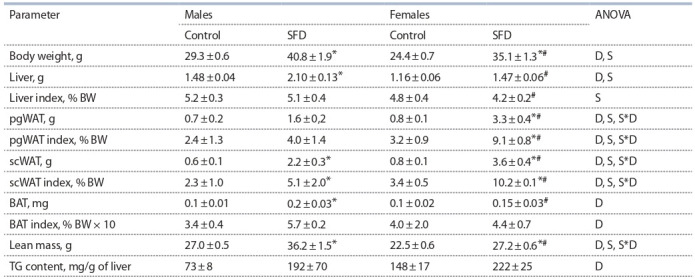
Weight-related parameters in mice, fed standard chow (control) and sweet-fat diet BW – body weight, two-way ANOVA was used with the factors S, sex effect; D, diet effect; and D*S, interactive effect of sex and diet. * p < 0.05 versus control group, # p < 0.05 versus males in the same group by post-hoc Tukey test

In females, hepatic weight and index were lower than in males (P < 0.001 for both parameters). Consumption of SFD increased hepatic weight (P < 0.001), but did not affect its relative weight in males and females. Maximum sex differences in absolute and relative hepatic weight were manifested only under SFD-induced obesity. An increase in liver mass was as sociated with an increase in hepatic fat deposition: the content of triglycerides (TG) in the liver, increased upon consumption of SFD (P < 0.05) in mice of both sexes

of SFD (P < 0.05) in mice of both sexes. In females, the mass and index of pgWAT were higher than in males (P < 0.01 for both parameters). SFD consumption increased them (P < 0.001 for both parameters) largely in females than in males (interaction of factors P < 0.01 for both parameters) and contributed to the maximum manifestation of sex differences. 

The mass and proportion of scWAT in females were higher than in males (P < 0.05 and P < 0.001 respectively). Consumption of SFD increased the scWAT mass and index (P < 0.001 for both cases) largely in females than in males (interaction of factors P < 0.07 for weight and P < 0.01 for index) and contributed to the manifestation of significant sex differences.

In the control group, the BAT weight in males and females did not differ. SFD increased the BAT weight and index (P < 0.01 and P < 0.05 respectively), however, the increase, in contrast to the SFD effect on the pgWAT weight, was significantly more pronounced in males than in females and was statistically significant. As a result, the BAT weight in females was significantly lower than in males only under the SFD (P < 0.05).

In females, the lean mass was significantly lower than in males (P < 0.001). The consumption of SFD increased lean mass in mice of both sexes (P < 0.05), but in males largely (interaction of factors P < 0.001), thereby enhancing the expression of sex differences. 

Plasma metabolite and hormone levels

**Fig. 1.**
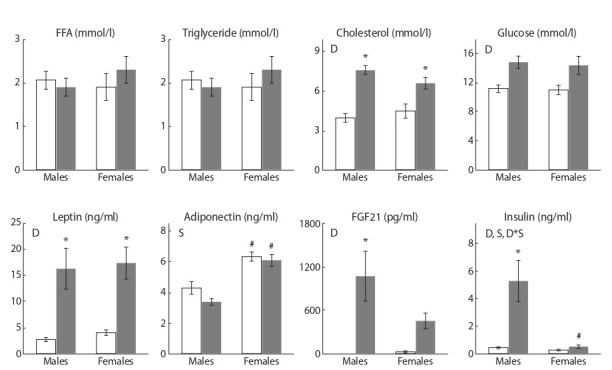
Serum biochemical parameters in mice, fed standard chow (control, white columns) and sweet-fat diet (grey columns). Two-way ANOVA was used with the factors S, sex effect; D, diet effect; and D*S, interactive effect of sex and diet. * p < 0.05 versus control group, # p < 0.05 versus males in the same group by post-hoc Tukey test.

In females, blood insulin levels were lower and adiponectin levels were higher than in males (P < 0.05 for insulin and P < 0.001 for adiponectin) in both groups (Fig. 1). SFD consumption increased blood levels of glucose, insulin, cholesterol, fibroblast growth factor (FGF21), and leptin (P < 0.01 for glucose, insulin, FGF21 and P < 0.001 for cholesterol and leptin) and did not alter the levels of free fatty acids (FFA), TG and adiponectin in mice of both sexes. Sex dimorphism was revealed only in the response of insulin and FGF21 to the SFD. Plasma insulin concentrations increased only in males and did not change in females, as evidenced by the significant interaction of factors sex and diet (P < 0.05). Plasma FGF21 concentration also significantly and reliably increased only in SFD males, while in SFD females the increase was less pronounced and not significant.

Glucose tolerance and insulin tolerance tests

**Fig. 2.**
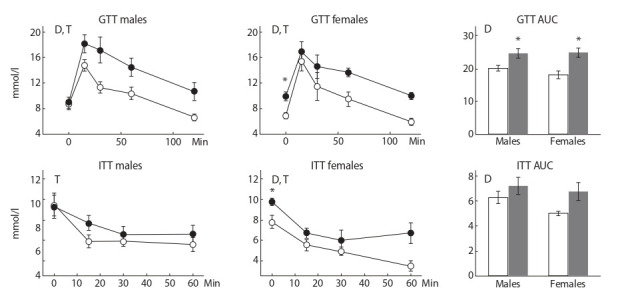
Blood glucose level and area under the curve (AUC) in GTT and ITT in mice, fed standard chow (control, white symbols) and sweet-fat diet (black symbols). Three-way ANOVA with factors sex, diet, and time (minutes 0, 15, 30, 60, 120 for GTT and 0, 15, 30, 60 for ITT) was used. T, time effect, and D, diet effect. * p < 0.05 versus control group by post-hoc Tukey test.

In control males, insulin sensitivity was lower than that of control females. SFD consumption reduced glucose tolerance and insulin sensitivity in both males and females (P < 0.001 in all cases) (Fig. 2). However, the effect of the SFD was more pronounced in females: the fasting blood glucose level and the glucose excretion curve in the ITT in the SFD females were higher than in the control ( p < 0.05 in both cases), while in the SFD males these parameters did not differ from the control.

Gene expression in metabolic tissues

**Fig. 3.**
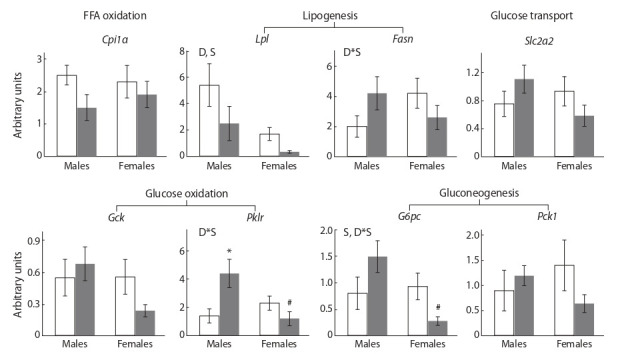
The mRNA levels of hepatic genes involved in glucose and lipid metabolism in mice, fed standard chow (control, white columns) and sweet-fat diet (grey columns). Two-way ANOVA was used with the factors S, sex effect; D, diet effect; and D*S, interactive effect of sex and diet. * p < 0.05 versus control group, # p < 0.05 versus males in the same group by post-hoc Tukey test.

Among the studied hepatic genes, only Lpl expression was dependent on sex (P < 0.01): it was lower in females than in males. The consumption of SFD down regulated the expression of this gene regardless of sex (Fig. 3). The consumption of SFD was accompanied by sex-dependent changes in the expression of the Fasn (fatty acid synthesis), G6pc (gluconeogenesis), and Pklr (glycolysis) genes: SFD males showed increased, while SFD females – decreased the mRNA levels of these genes in relation to control (interaction of factors P < 0.05 for all genes). As a result, in SFD females, G6pc gene expression was fivefold and Pklr gene expression was 2.4 times lower than in SFD males (P < 0.05).

**Fig. 4.**
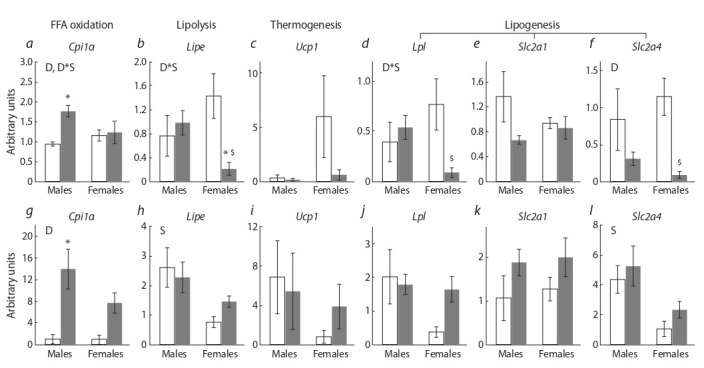
The mRNA levels of pgWAT (a–f) and scWAT (g–l) genes involved in lipid metabolism in mice, fed standard chow (control, white columns) and sweet-fat diet (grey columns). Two-way ANOVA was used with the factors S, sex effect; D, diet effect; and D*S, interactive effect of sex and diet. * p < 0.05 versus control group, $ p < 0.05 versus males in the SFD group by Student’s test

There were no sex differences in the expression of the studied genes in pgWAT (Fig. 4, a–f  ). The consumption of SFD influenced the expression of Cpt1α (fatty acid oxidation), Lipe (lipolysis), and Lpl (lipogenesis) genes differently in males and females (interaction of factors p < 0.05 in all cases): only in males, Cpt1α mRNA level increased, only in females, Lipe and Lpl mRNA levels decreased. SFD down regulated Slc2a4 gene expression regardless of sex (P < 0.01). However, the decrease was more pronounced in females (12 times) than in males (2.7 times).

In scWAT, in contrast to pgWAT, sex influenced the expression of the Lipe and Slc2a4 genes (P < 0.05 for Lipe, P < 0.01 for Slc2a4): it was lower in females than in males (see Fig. 4, g–l ). SFD consumption did not affect the expression of most of the studied genes and only upregulated Cpt1α gene expression of (P < 0.001). In males, this increase was significant (P < 0.01 post-hoc Tukey test) and more pronounced (13 times) than in females (7 times). 

**Fig. 5.**
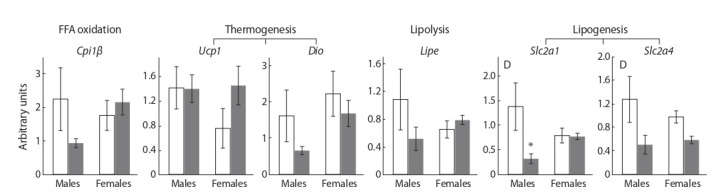
. The mRNA levels of BAT genes involved in lipid metabolism in mice, fed standard chow (control, white columns) and sweet-fat diet (grey columns). Two-way ANOVA was used with the factors S, sex effect; D, diet effect; and D*S, interactive effect of sex and diet. * p < 0.05 versus control group by post-hoc Tukey test.

There were no sex differences in the expression of the studied genes in BAT (Fig. 5). SFD did not affect the expression of genes involved in fat metabolism (Cpt1β, Lipe) and thermogenesis (Ucp1, Dio2); however, it down regulated the expression of genes that control glucose uptake into the cell – Slc2a1 and Slc2a4 (P < 0.05 for both genes).

Diet had a sex-independent effect on Slc2a4 – and sexdependent on Slc2a gene expression (interaction of factors P = 0.06, tendency). Diet reduced Slc2a1 mRNA level by 4.5 times only in males, as a result, its expression in SFD males was 2.5 times less than in SFD females.

## Discussion

A sweet-fat diet increases fat and carbohydrate proportion in food. To maintain a constant levels of blood lipids and carbohydrates, two ways of adaptation are possible: the deposition of fat excess and increased glucose and fatty acid oxidation in the liver, muscles and adipose tissues. Our results suggest that in male mice, both ways of adaptation were used and in female mice, the reservation of energy excess in the form of white fat prevailed. SFD males showed increased scWAT weight, although to a lesser extent than SFD females, and increased fatty acid oxidation in WAT and glucose in the liver. Only SFD males demonstrated increased expression of Cpt1α gene (a marker of fatty acid oxidation) in white adipose tissue and Pklr gene (a marker of glucose oxidation) in the liver. In addition, SFD males showed a more pronounced, than SFD females increase in “lean mass”, which may indicate a more intensive oxidation of metabolic substrates that occurs in the muscles, and, possibly, a greater infiltration of fat into muscle tissue.

The phenomenon of more intense fat accumulation in females than in males when fed high-energy diets was previously described in the literature (Priego et al., 2008; Medrikova et al., 2012; Chang et al., 2018). Several physiological mechanisms of this phenomenon have been proposed. First, estradiol is known to increase the number of adipocyte progenitor cells (Dieudonne et al., 2000); therefore, their number is higher in females than in males (Wu et al., 2017; Chang et al., 2018). Second, SFD increases the number of adipocyte progenitor cells only in females (Wu et al., 2017; Chang et al., 2018), but the reason for this is not known. Third, insulin sensitivity and lipogenesis are increased in white fat adipocytes in females compared to males (Macotela et al., 2009).

The data on pgWAT genes expressions obtained in our work complement the known mechanisms of intensive fat accumulation in females under sweet-fat diet consumption. In pgWAT, only in females, diet downregulated expression of genes, involved in lipid metabolism – Lipe (lipolysis) and Lpl (lipogenesis). Expression of the Slc2a4 gene, which is also involved in lipogenesis, was reduced in SFD females to a much greater extent than in SFD males. Recently we demonstrated, that SFD reduced mRNA level of Pparγ (a transcription factor, the main regulator of adipocyte differentiation and function) in pgWAT, only in females (Bazhan et al., 2019). Together, these data suggest that a decrease in the expression of genes involved in the regulation of multidirectional processes in pgWAT, is an indicator of a decrease in the intensity of lipid metabolism, what can contribute to the conservation of energy in the form of white fat reserves in females.

SFD increased the Cpt1α gene expression in WAT of males, regardless of localization, which is consistent with the literature data (Warfel et al., 2017). The mechanism of selective activation of the Cpt1α gene expression in WAT of males fed high-energy diets is not known. In our work, increased expression of the Cpt1α gene in WAT of SFD males was associated with a multiple increase in the FGF21 blood level. Previously, we and other authors have shown that, selectively in males, FSD increased not only the blood FGF21 levels, but also its gene expression in the liver (Chukijrangroat et al., 2018; Gasparin et al., 2018; Bazhan et al., 2019). Apparently, the activation of the FGF21 system in males was much more pronounced than in females upon SFD consumption.

The liver is the main site of FGF21 synthesis, and adipose tissues are the main site of FGF21 action. In pharmacological and genetic studies, FGF21 has been shown to increase energy expenditure in WAT and BAT and insulin sensitivity at the whole body level (Xu et al., 2009; Zhang, Li, 2014). These effects may be due to FGF21 facilitates oxidative processes in WAT mitochondria (Chau et al., 2010), in particular by stimulating the expression of the Cpt1α gene (Coskun et al., 2008). It can be assumed that the increased Cpt1α gene expression in WAT of SFD males contributed to the increased fatty acid oxidation and prevented fat deposition. Therefore, pgWAT and scWAT weights in SFD males were significantly less than in SFD females. 

The liver plays a crucial role in the regulation of energy homeostasis at the level of the whole body and is the main site of estradiol action in the regulation of insulin sensitivity. According to our results, it is also the central link in the implementation of various pathways of adaptation to SFD in male and female mice: the response to SFD of most studied hepatic genes was sex-dependent. The mRNA levels of the Fasn, Pklr, G6pc, and Slc2a2 genes were increased or unchanged, relative to control, in SFD males, and were decreased in SFD females.

SFD males showed increased or unchanged expressions of Fasn, Pklr, G6pc, and Slc2a2 genes, while SFD females showed decreased expressions of these hepatic genes. The same multidirectional dynamics of the transcriptional response to SFD were observed for other hepatic genes measured in our work, although the sex effect was not statistically significant. These results are in good agreement with the previously published data showing that only in male mice, SFD increases the hepatic expression of the peroxisome proliferator-activated receptor-α (PPARα), a transcription factor that enhances the expression of many hepatic genes involved in the regulation of carbohydrate-lipid metabolism (Gasparin et al., 2018; Bazhan et al., 2019; Sasaki et al., 2019). As a result, the expression of these genes (Slc2a2, Gck, Pklr, G6P, and Pck1) was lower in females than in males under SFD-induced obesity. Taken together, our data suggest that male mice respond to SFD with enhanced oxidation of glucose and fatty acids not only in WAT, but also in the liver.

It is possible that the mechanism of selective FGF21 activation in males with SFD-induced obesity was associated with hyperinsulinemia, which was revealed in our work and in the works of other authors, carried out on rodents consuming high-calorie diet (Rodríguez et al., 2003; Priego et al., 2008). An association was found between high plasma insulin and FGF21 levels in obese rodents and humans (Zhang et al., 2008; Chavez et al., 2009), the exact mechanism of which is unknown. It can be assumed that the increased blood FGF21 levels in SFD males counteracts the development of metabolic syndrome: FGF21 reduce body weight, normalize the lipid profile, and increase insulin sensitivity in various models of insulin resistance (Zhang, Li, 2014). In females, the SFD consumption caused a less pronounced than in males and insignificant increase in the blood insulin and FGF21 levels; apparently, FGF21 did not participate in adaptation to the SFD in females.

Our results showed that the SFD consumption stimulated the development of metabolic syndrome regardless of sex: obesity, increased blood glucose, insulin, cholesterol levels, hepatic TG content, and decreased glucose tolerance and insulin sensitivity. It should be noted that the SFD consumption disturbed different links in the regulation of blood glucose levels in males and females: satiated hyperinsulinemia was observed only in SFD males, and fasting hyperglycemia – only in SFD females. The mechanisms of sex-associated dysregulation of carbohydrate metabolism under obesity caused by a sweet-fat diet consumption are not known and need to be explored.

In BAT, in contrast to WAT, glucose enters the cells through Glut1 to the same extent as through Glut4 (Czech, 2020). The regulation of the expression of these genes and corresponding protein activity in BAT differs from that in WAT. Slc2a4 gene expression is regulated by insulin (Burcelin et al., 1993), and gene expression and activity of the Glut1 protein are regulated by norepinephrine through activation of beta 3 adrenoreceptors via a cAMP-dependent mechanism (Cannon, Needergaart, 2004). Our data demonstrated that in BAT, SFD consumption reduced the Slc2a4 gene expression equally in males and females, and the Slc2a1 gene expression only in males. The latter may be due to the effect of sex on the expression of beta3-adreno receptors under SFD consumption. The cafeteria diet has been shown to reduce the level of protein and the beta3-adreno receptor gene expression in BAT in male rats, but does not affect them in female rats (Rodríguez et al., 2001).

Glucose itself is not the dominant thermogenic substrate in BAT, it is converted into fatty acids, which oxidizing in the mitochondria, enhance thermogenesis (Cannon, Needergaart, 2004). It has been shown that obesity caused by long-term FSD consumption is associated with a decrease in energy consumption at the level of the whole body and with a decrease in thermogenesis at the level of BAT (Penna-de-Carvalho et al., 2014). It can be assumed that diet-induced decrease in the expression of glucose transporter genes in BAT will be accompanied by a decrease in thermogenesis, and this effect will be more pronounced in males than in females. This assumption is supported by data obtained earlier that in male mice, high-energy diets reduces in BAT, the expression of transcription factor Pparγ which stimulates the expression of target genes involved in the regulation of thermogenesis (Penna-de-Carvalho et al., 2014; Bazhan et al., 2019).

## Conclusion

Thus, the results showed that in mice, adaptation to the consumption of SFD associated with the accumulation of excess white fat was observed both in males and females, but in females to a much greater extent than in males. In females, the diet down regulated the expression of hepatic and white adipose tissue genes involved in carbohydrate and fat metabolism, which could contribute to a decrease in energy expenditure and white fat accumulation. Only in males, adaptation to SFD, associated with enhanced oxidation of energy carriers in the liver and white fat, was observed, SFD males showed a significantly increased lean mass, blood insulin and FGF21 levels, and expressions of the Cpt1α genes in white fat tissues and Pklr in the liver. This suggests increased energy expenditure for fatty acid and glucose oxidation in WAT, muscle, and liver, and may inhibit the storage of energy in the form of white fat.

Adaptation ensure the maintenance of constant FFA and triglyceride blood levels, but led to the appearance of signs of insulin resistance (decreased insulin sensitivity, glucose tolerance, and increased TG levels in the liver) in males and females. Consumption of SFD disrupted different links in the regulation of insulin sensitivity in males and females: only in males, it caused satiated hyperinsulinemia and only in females – fasting hyperglycemia. The study of the sex characteristics of the molecular physiological mechanisms underlying adaptation to SFD in mice is a necessary step for the development of a gender-specific approach to the correction of metabolic disorders in humans.

## Conflict of interest

The authors declare no conflict of interest.
